# Supplementary material for: Impact of SETD8/KMT5A overexpression on hepatocellular carcinoma progression and prognosis
Source: PLoS One. 2026 Apr 13;21(4):e0337503. doi: 10.1371/journal.pone.0337503 (PMC13075679; doi:10.1371/journal.pone.0337503)
Supplement: S1 Table — (DOCX) [file pone.0337503.s001.docx]

Supporting information 1: Primer sequences for quantitative qRT-PCR

| **siRNA** |  | **Sequence** |
| --- | --- | --- |
| siEGFP | Sense | 5’ GCAGCACGACUUCUUCAAG 3’ |
|  | Antisense | 5' CUUGAAGAAGUCGUGCUGC 3' |
| siSETD8#1 | Sense | 5' CUGCGUUUAUAAACCAUGA 3' |
|  | Antisense | 5' UCAUGGUUUAUAAACGCAG 3' |
| siSETD8#2 | Sense | 5' GUGUCAACUGGUCGAGAUA 3' |
|  | Antisense | 5' UAUCUCGACCAGUUGACAC 3' |
