# Supplementary material for: Impact of SETD8/KMT5A overexpression on hepatocellular carcinoma progression and prognosis
Source: PLoS One. 2026 Apr 13;21(4):e0337503. doi: 10.1371/journal.pone.0337503 (PMC13075679; doi:10.1371/journal.pone.0337503)
Supplement: S2 Table — (DOCX) [file pone.0337503.s002.docx]

Supporting information 2

| **Gene name** | **Primer sequence** |
| --- | --- |
| GAPDH (housekeeping gene) - f | 5' GCAAATTCCATGGCACCGTC 3' |
| GAPDH (housekeeping gene) - r | 5' TCGCCCCACTTGATTTTGG 3' |
| SETD8 - f | 5' TCTTGTGATTCCACCAATGCAG 3' |
| SETD8 - r | 5' CCTTCGGACAGGGTAGAAATCC 3' |
